# Secondary school students’ perception of the online teaching experience during COVID‐19: The impact on mental wellbeing and specific learning difficulties

**DOI:** 10.1111/bjep.12475

**Published:** 2021-12-13

**Authors:** Thomas Walters, Nicola J. Simkiss, Robert J. Snowden, Nicola S. Gray

**Affiliations:** ^1^ Howell’s School Cardiff UK; ^2^ Department of Psychology Swansea University UK; ^3^ School of Psychology Cardiff University UK; ^4^ Caswell Clinic Swansea Bay University Health Board Bridgend UK

**Keywords:** concentration, COVID‐19, online learning, specific learning difficulties

## Abstract

**Background:**

Student engagement and concentration is critical for successful learning. Due to the COVID‐19 pandemic, there has been a dramatic increase in the use of online learning which may affect engagement and concentration, particularly for those students with specific learning difficulties.

**Aims:**

1. Students would show lower scores on all the measures of student experience when judging these during online learning versus learning within the classroom.
2. This negative impact of online learning on concentration, engagement, perceived learning, and self‐worth compared to classroom education would be more significant for those with specific learning difficulties.
3. The drop in student experience scores due to online learning would be associated with poorer mental well‐being.

**Sample:**

Four hundred seven pupils aged 11–18 years at a secondary education school in Wales.

**Methods:**

A retrospective online survey comparing pupils’ normal classroom experience to learning online during the first national lockdown in the United Kingdom (March–July 2020).

**Results:**

Pupils’ learning experiences (concentration, engagement, ability to learn, and self‐worth from learning) were significantly lower for online learning compared to the classroom learning. These differences were more marked in students with specific learning difficulties. Perceived ability to learn and engage during classroom and online learning were also associated with mental well‐being.

**Conclusions:**

The move to online learning appears to have affected students’ ability to concentrate and engage in their schoolwork and appears to have reduced their ability to learn and get self‐worth from their work. These decreases are associated with a decrease in mental well‐being. The effects appear to be exacerbated in some students with specific learning difficulties.

## Background

As with many aspects of everyday life, COVID‐19 has had a severe impact on education worldwide (Onyema et al., 2020). On the 23rd of March 2020, the World Health Organization declared a global health emergency, resulting in schools across the United Kingdom physically closing (Toquero, 2020) and moving to online learning (Friedman, 2020). While schools remained open to those particularly vulnerable and the children of key workers, lessons were generally still delivered online as many teachers could not be present in schools. For those pupils in school, classes were often mixed, supervised by available staff, with students often completing work individually and not in class groups. Distance learning is likely to have consequences on the students’ educational experience for many reasons, including home distractions, less effective supervision, and limited interaction with peers.

In this study, we examined perception of secondary school students’ educational experience online compared to their usual classroom experience to understand the problems associated with online learning as experienced during the COVID‐19 in the United Kingdom. The situation during the pandemic was unprecedented and hopefully not to be repeated. However, it provides an opportunity whereby schools may use online learning methods more frequently, perhaps as an adjunct to in‐person teaching. In contrast to teaching that is planned and designed to be online, the online learning measured in this study was a temporary shift of instructional delivery to an alternate delivery mode due to the current situation. It involves the use of fully remote teaching solutions for instruction or education that would otherwise be delivered face‐to‐face. It is important to highlight that the online learning methods that were utilized by schools were unplanned, last‐minute and with very little support or experience in this area from current schoolteachers which is not fully comparable as a planned process of online learning.

We focussed on pupils’ learning experience, measuring perceptions of concentration, engagement, ability to learn, and self‐worth from learning. We further examined if having specific learning difficulties was associated with greater perceived problems. We have also taken measures of pupils’ current mental well‐being to explore whether difficulties in concentration, engagement, and learning are associated with mental well‐being.

### Online versus classroom‐based learning

In recent years, online technology has noticeably transformed learning and teaching environments (Ni, 2013). The debate over online learning’s ability to replace the face‐to‐face education and teacher–student relationship remains unresolved (Schmid et al., 2014). Classroom activities are important beyond education and knowledge acquisition and help students acquire social skills that have implications for future personal and professional growth (Goodman et al., 2015). Interaction with teachers and other students is essential for developing positive self‐esteem, self‐confidence, and also improving students’ ability to work collaboratively and productively with peers (de Souza Fleith, 2000).

A common concern surrounding online learning is the absence of face‐to‐face interaction (Bao, Selhorst, Moore, & Dilworth, 2018). Fraser and Goh (2003) noted that communication behaviours encouraged in a face‐to‐face classroom are not always supported or available within online teaching. The ability to ask questions, share opinions, or disagree with points is fundamental to learning (Chin & Osborne, 2008).

Research has often compared performance and learning outcomes due to online teaching versus classroom‐based teaching (Akkoyunlu & Soylu, 2008; Ni, 2013). Kemp and Grieve (2014) compared undergraduate students’ preferences and academic performance during the presentation of class material and written assessments online and within the classroom. Students rated face‐to‐face teaching much higher than online teaching and feedback suggested they felt more engaged during face‐to‐face teaching due to receiving immediate feedback. However, despite preference for in‐class teaching, there were no significant differences in the students’ academic performance between the two modes.

Multiple studies have explored online student engagement in higher education (Jeffrey, Milne, Suddaby, & Higgins, 2014), but few studies have explored online learning at school levels (Al‐Salman, 2011). Friedman (2020) looked at students’ online learning challenges amid the pandemic, using a quantitative survey design to determine distractions students face when studying online. South Korean high school students (ages 15–19 years) highlighted that their most significant challenge was staying awake and focused during online classes, followed by distractions such as watching online videos, rather than engaging in online lessons. Students also reported misunderstanding instructions and limited feedback. This research highlights vital challenges to students during online teaching from a student’s perspective.

There is increasing research on online learning due to the current pandemic, with most research canvassing teachers’ or parents’ perspectives. Garbe, Ogurlu, Logan, and Cook (2020) collected data from parents who had a child who had moved from face‐to‐face teaching to learning online in spring 2020. Using thematic analysis, researchers identified several critical themes (e.g., a lack of learning motivation). Parents believed that pupils’ lack of motivation during online learning was due to a lack of a teacher’s presence. Further to this, they thought children were uncomfortable using computer screens, recording themselves and generally preferred face‐to‐face learning.

Pupils’ perception of their engagement and concentration is an essential component of all teaching and learning (Dixson, Greenwell, Rogers‐Stacy, Weister, & Lauer, 2017). Parental and teacher perspectives can provide useful insights. However, using pupils’ self‐report may give a clearer understanding of the challenges and personal experiences. Recent research by Chopra et al. (2021) explored the experiences and perceived impact of the COVID‐19 lockdown among adolescents in England. Using thematic analysis, they explored the self‐care and coping strategies of young people and found four themes: change, embracing lockdown, loss, and stress. Further to this, Ashworth highlights how during the transition to online learning young people felt over time they also began to miss time with their peers and being within the school environment and some reported finding it a struggle to complete tasks without a structure. In addition to this, Yates, Starkey, Egerton, and Flueggen (2021) also looked at high school students experiences during COVID‐19 through qualitative and quantitative questionnaires. They also found pupils struggled with motivation to study due to a lack of extrinsic drivers of a school routine. The current research is interested in a self‐report approach to teaching and learning and how this was affected by the shift to online learning, rather than focusing on well‐being.

### Specific learning difficulties

Specific learning difficulties are defined by the Individuals with Disabilities Education Improvement Act of 2004 as a disorder in one or more of the basic psychological processes involved in physical or sensory needs that manifests itself in difficulty to listen, think, speak, or complete mathematical calculations (Yell, Shriner, & Katsiyannis, 2006).

There is limited information on whether the online learning effects outlined above are greater in pupils with specific learning difficulties (Erickson, Trerise, VanLooy, Lee, & Bruyère, 2009). Online experiences may be increasingly difficult among those with specific learning difficulties.

For pupils with dyslexia, particular study skills are identified as problematic. Woodfine, Nunes, and Wright (2008) sought to address difficulties in learning dyslexia in synchronous e‐learning environments among higher education students. Through problem‐solving and qualitative interviews, they found text‐based synchronous learning activities isolated and demotivated students with dyslexia. Students with dyslexia fell behind other students, often being slower to read a text and needing more time to complete tasks.

Similarly, research focusing on children with low versus high working memory in classroom learning has shown low working memory leads to poorer performance in mathematics and reading (Kyttälä, 2008). Thus, it would seem probable that the shift to online learning might be disadvantageous for such students (Fellman, Lincke, Berge, & Jonsson, 2020).

The effects of working memory problems on learning are an area of current focus within educational research. The common acceptance that working memory has limited capacity suggests instructional methods should avoid overloading this capacity (Sweller, Van Merrienboer, & Paas, 1998). More recently, this has been extended to consider how educational technology could limit extraneous load on working memory (Sweller, 2020). This is particularly important with pupils with working memory difficulties and dyslexia (Smith‐Spark & Fisk, 2007).

Success in online learning is strongly dependant on students’ engagement with course content (Martin & Bolliger, 2018). Successful online learning requires self‐regulation, time management, and organization (Kauffman, 2015). Such skills are difficult for many students, including students with processing speed impairments (Jarrold, Mackett, & Hall, 2014). It is important to consider difficulties with engagement that students with specific learning difficulties can face when transferring between learning environments.

### Mental well‐being

Schools are an essential source of health and mental health support (Hoffman & Miller, 2020). Early evidence indicates that adolescent mental health and well‐being are suffering during the pandemic with increased rates of anxiety and lower quality of life (Ravens‐Sieberer et al., 2021). However, there is limited research on how the shift to online learning has affected pupils’ mental health.

Traditional classroom‐based education and health are closely linked (Bradley & Greene, 2013). Research has often shown school engagement affects mental well‐being (Bond et al., 2007; Hakanen & Schaufeli, 2012). School engagement is influenced by factors involving reading, writing skills and the school context, such as participation in lessons and support (Jennings, 2003). We aim to determine whether the ability to concentrate and engage in the online learning environment would be predictive of current mental well‐being.

### Research objectives

Student engagement and concentration are critical for successful learning (Appleton, Christenson, & Furlong, 2008); hence, understanding changes in these factors due to moving education online is important. This study explores students’ perceptions of the online and classroom‐based teaching experience during COVID‐19. By comparing the two learning contexts, conclusions can be made about students’ ability to engage, concentrate, learn, and experience self‐worth. To our knowledge, this study provides the first explicit comparison of the perceptions of online learning with classroom learning in secondary school children and how this is associated with mental well‐being and particularly specific learning difficulties. We examined whether there are differences in concentration, motivation, and engagement from a pupil’s perspective during online and classroom‐based teaching and address whether this difference has a more substantial impact on children with specific learning difficulties.

Our main hypotheses were:

1. Students would show lower scores on all the student experience measures (concentration, engagement, perceived learning, and self‐worth from learning) when judging these during online teaching versus teaching within the classroom.
2. This negative impact of online learning on concentration, engagement, perceived learning, and self‐worth compared to classroom education would be more significant for those with specific learning difficulties.
3. The drop in student experience scores due to online learning would be associated with poorer mental well‐being.

## Method

### Participants

All 462 pupils at a secondary education school in Wales were invited to participate in the survey. The school is an all‐girls secondary school with co‐education in Years 12 and 13 (age 16–18). A total of 407 pupils completed the survey (17 males, 390 females). Participants were aged between 11 and 18 years, of which (34 pupils in Year 7, 68 pupils in Year 8, 56 pupils in Year 9, 59 pupils in Year 10, 63 pupils in Year 11, 69 pupils in Year 12 and 53 pupils in Year 13, five pupils selected prefer not to say. Of the sample, we had data on the number of pupils with specific learning difficulties due to dyslexia (*n* = 23), dyspraxia (*n* = 3), autism spectrum disorder (ASD) (*n* = 3), attention deficit hyperactivity disorder (*n* = 1), hearing impairment (*n* = 2), visual impairment (*n* = 1), working memory problems (*n* = 29), and processing speed problems (*n* = 51). The identification of specific learning difficulties was reliant on the school’s existing data. Specific learning difficulties were pupils identified by the school as having an identified need, either by identification through formal testing by an Educational Psychologist or specialist teacher assessor with a current Specific Learning Difficulties Assessment Practising Certificate, or by having a Statement of Special Educational Needs. Due to the complexity of the conditions and comorbidity, a specific learning difficulty such as dyslexia will potentially also have processing speed problems or working memory problems; conversely, those with issues with processing speed problems or working memory problems may have dyslexia without formal diagnosis.

The population of the school determined the sample sizes. Overall, the sample size (*n* = 407) produced a powerful test (>99%) of the main hypotheses that online learning would produce more negative ratings on our dependent variables even for small effect size (*d* = .20; *α* = .05 – see Cohen, 1988). The small sample sizes constrained our secondary hypotheses relating to specific learning difficulties, and only those groups with a sample size > 25 were analysed. For the smallest group (working memory group, *n* = 29) against a much larger control group and using standard estimates for a ‘medium’ effect size (*d* = .50; *α* = .05) gives a power of 73.5%, which rises to 91.5% for the processing speed problem group (*n* = 51).

### Design

A quantitative retrospective design was used. Pupils completed the survey during the period of 9/11/2020–27/11/2020 when schools had re‐opened.

### Procedure

Pupils were asked to compare their normal classroom experience to learning online as they had experienced this for the period of home learning during lockdown in Wales (23/3/2020–17/7/2020). Pupils watched a brief video explaining study aims and objectives. The survey was completed during the morning registration class and took approximately 15 min to complete. An online survey platform (Survey Monkey) allowed for convenient data collection and easy survey distribution to each pupil (Symonds, 2011).

Following completion, data were downloaded by the senior leadership team at the school. Information on specific learning difficulties was added to the database. The database was anonymized before data analysis.

### Survey development

Following a review of previous literature on the impact of online education, an initial pool of survey items was collated covering concentration, engagement, and motivation. Motivation items later became ‘Ability to learn’ and ‘self‐worth from learning’. Having an ability to learn and self‐worth covers a wider construct of motivation, opportunity, and ability (Dahlin, Chuang, & Roulet, 2018). Once the questionnaire's content was decided, it was then deemed necessary that the language was adapted for use with young people. Each item was piloted on six children aged between 10 and 17 years. Questions were written clearly and understandably to avoid any ambiguity for pupils (Bell, 2007). 10–15 min is the recommended completion time for 11‐year‐olds (Rea & Parker, 2014) as concentration and reluctance to complete the survey can impact the data quality. The senior leadership team at the school reviewed the final version of survey for suitability for the age group to be studied (age 11–18) and readability.

### Measures

The survey comprised seven sections. The first section was an information page outlining the research aims. The following pages obtained demographic information, including name and year group. This information was used by the school to link survey data with information on specific learning difficulties and then destroyed to retain anonymity.

#### Classroom versus Online Study Questionnaire (COSQ)

The main survey, which we term the Classroom versus Online Study Questionnaire (COSQ), consisted of four sets of questions (Table 1). For each question on the COSQ, pupils were asked to respond to a 4‐point Likert scale 1 (*Not at all*) to 4 (*A great deal*) separately for perceptions of learning in the classroom and during online education. Negatively worded items were reversed scored in summing of the subscale scores. High scores indicate more positive experiences (i.e., better concentration and better self‐worth from learning).

**Table 1:** Classroom versus Online Study Questionnaire (COSQ)

	Not at all	A Little	A lot	A great deal
*Concentration when learning*				
1. I find it difficult to concentrate on the lesson (r)				
2. Lessons make me tired (r)				
3. I get distracted by things around me (r)				
4. I am able to focus on the lesson				
5. I can cope well with the material presented in class				
6. I get distracted from my learning in the classroom (r)				
*Engagement and interest in learning*				
7. I enjoy learning				
8. I try hard in my lessons				
9. Lessons are interesting				
10. Lessons are enjoyable				
11. I do look forward to lessons				
12. I feel motivated to engage in lessons				
*Self‐worth from learning*				
13. I feel competent (capable) in lessons				
14. I understand lessons quickly and easily				
15. I believe in myself and my ability to learn				
16. I worry I am going to fail in tests (r)				
17. I feel confident that I am doing well in school				
18. I believe I am falling behind in my learning (r)				
19. I feel anxious when trying to keep up with my learning (r)				
*Ability to learn*				
20. I find it difficult to follow instructions (r)				
21. Members of my class help me to understand				
22.I believe in myself and my ability to learn				
23. I have enough time to think				
24. New ideas are clearly explained				
25. I am able to go over lessons when I am unsure				
26. I can ask for help when I need to				
27. I keep up to date with my work				
28. I feel rushed during the lesson (r)				
29. Being together with my class is important to my learning				
30. I can hear the teacher clearly				
31. I can see the presentations (such as PowerPoint) or whiteboard clearly				
*Other*				
32. Is there anything else that effects your online learning that you would like to make us aware of?				
*(r) = reversed scored items				
*Additional question*				
For both online and classroom, If you get distracted, please tick any that apply				
I do not get distracted Friends Family Pets Devices, such as phones and computers Noises outside Distracting thoughts Other (Please specify)				

Note

Participants complete items for both in the classroom and online learning.

##### Concentration

Concentration is a state of mental alertness and focused activity (Posner & Petersen, 1990). This section included six questions (e.g., *I find it hard to concentrate on the lesson*). The internal reliability (defined via Cronbach alpha (α)) for this scale was high (α = .84 for online and α = .78 for classroom learning).

Pupils were also asked ‘If you get distracted, please tick any that apply’ and were provided with a list of six distractions (e.g., devices, such as phones and computers).

##### Engagement

This construct was measured using items that covered pupils’ engagement, motivation, and interest during lessons. This section included six questions (e.g., *I feel motivated to engage in lessons)*. Internal reliability was high for this scale (α = .89 for online and α = .91 for classroom learning).

##### Ability to learn

This construct was measured using items that covered pupils’ perceptions of their ability to learn. This section included 12 questions. (e.g., *I believe in myself and my ability to learn*). Internal reliability was high for this scale (α = .80 for online and α = .83 for classroom learning).

##### Self‐worth from learning

Self‐worth refers to the overall appraisal of one’s worth (Harter, 2006). This section aimed to measure perceptions of self‐worth from learning. It included seven questions (e.g., *I believe in myself and my ability to learn*). Internal reliability was high *(*α = .86 for online and α = .85 for classroom learning).

Finally, pupils were asked ‘Is there anything else that affects your online learning that you would like to make us aware of?’.

#### Mental well‐being

The Short Warwick Edinburgh Mental Wellbeing Survey (SWEMWBS) (Stewart‐Brown et al., 2009) was used to measure mental well‐being by asking pupils how often over the past two weeks they had been: (e.g., *feeling optimistic about the future;* and *I’ve been dealing with problems well*). Responses ranged from 1 (*none of the time*) to 5 (*all the time*) on a 5‐point Likert scale. SWEMWBS scores ranged from 7 to 35 with higher scores reflecting greater well‐being (Stewart‐Brown et al., 2009). Internal consistency of the SWEMWBS was high for the current sample (Cronbach α = .84).

### Ethical considerations

Ethics Committee approved the project, ref: 2020‐4935‐3931. Pupil's parents/carers received an email detailing the research aims. Parents were given the option to opt out at any time. Informed consent was also obtained online before completing the survey. Senior leaders at the school had access to the self‐report data to add information on specific learning difficulties and anonymize the data for the research team, which was stored in a secure database. Pupils and parents were informed that the school would collate the self‐report data, and then, the survey would be made anonymous before the data were analysed.

### Statistical analysis

Data were analysed using IBM SPSS Statistics (version 26). The distributions of all measures (including subscales) were examined for deviations from normality as recommended by Tabachnick, Fidell, and Ullman (2007). Data for each scale of the COSQ were calculated by adding the scores from each question. Missing responses were pro‐rated to the scale mean unless more than one question of the scale were missing, in which case the scale was regarded as invalid. For 407 pupils, within the SEMWBS (12.53% had at least 1 missing value), (Concentration had 8.84% had at least 1 missing value), (Engagement 7.37% had at least 1 missing value), (Self‐Worth‐ 9.58% had at least 1 missing value), (Ability to learn 11.56% had at least 1 missing value). Overall, 6.57% of questions were missed. A similar procedure was used for the SWEMWBS. All distributions were approximately normal (e.g., all Skewness and kurtosis were between −1 and +1), so parametric statistics were used throughout.

## Results

### Comparison of classroom versus online learning

Data are presented in Table 2. In line with our first hypothesis, each scale score was significantly lower for studying online than in the classroom (all *p*s < .001). The effect sizes were ‘large’ (Cohen, 1988) for the Concentration, Engagement, and Ability to Learn scales and ‘medium’ for the Self‐Worth scale.

**Table 2:** Descriptive statistics of the Classroom versus Online Study Questionnaire (COSQ)

	*N*		Classroom		Online		*p*‐value	Effect size (Cohen’s *d*) [95% CI]
		Possible range	Mean	*SD*	Mean	*SD*		
Concentration	379	(6–24)	19.3	2.8	16.2	3.7	<.001	0.94 [0.81, 1.08]
Engagement	384	(6–24)	18.2	3.8	14.9	4.1	<.001	0.82 [0.70, 0.94]
Ability to learn	377	(12–48)	37.2	5.5	32.5	5.9	<.001	0.83 [0.72, 0.95]
Self‐worth from learning	383	(7–28)	20.3	4.3	17.7	4.6	<.001	0.58 [0.50, 0.67]

### Distractions during classroom versus online learning

Information on the percentage of reported pupils’ distractions for online and classroom‐based learning are presented in Table 3. Overall, a higher percentage of pupils reported ‘no distraction’ (19.6%) in classroom learning than online learning (9.6%). Distraction by devices such as phones or computers was reported more during online learning (51.4%) than in classroom learning (14.4%). Similarly, distraction by family (37.9%) and pets (30.7%) were rated as frequent distractions for online learning. A high proportion of students rated ‘distracting thoughts’ as interfering with their concentration during both online learning (54.0%) and in the classroom (59.2%).

**Table 3:** Number of reported distractions for online and classroom learning (%)^1^

Distraction	Classroom		Online	
	Number	Per cent	Number	Per cent
*N*	382		385	
Not distracted	75	19.6	37	9.6
Friends	144	37.7	62	16.1
Family	0	0.0	146	37.9
Noise outside	134	35.1	187	48.6
Distracting thoughts	226	59.2	208	54.0
Pets	0	0.0	118	30.7
Devices	55	14.4	198	51.4
Other	27	7.1	33	8.6

^1^Pupils could tick all that applied, so the total number of distractions in the classroom and online is greater than the total number of participants.

### Relationship to specific learning difficulties

Our second hypothesis was that specific learning difficulties would be related to the impact of online learning for concentration, engagement, ability to learn, and self‐worth from learning. Given the low numbers for most of the groups with specific learning difficulties, only data relating to working memory problems (*n* = 29), and processing speed problems (*n* = 51) were analysed.

#### Working memory difficulties

Participants were grouped into those with working memory problems and students with no specific learning difficulties (control group). A two‐way mixed MANOVA showed a significant interaction between mode of delivery and group, *F*(4, 286) = 3.48, *p* = .009; Wilk’s Λ = 0.95; $\eta_{p}^{2}$ = .046.

At the univariate level, there was a significant interaction between mode of delivery and group for Concentration, *F*(1, 289) = 10.47, *p* = .001; $\eta_{p}^{2}$ = .035, and for Engagement, *F*(1, 289) = 4.57, *p* = .03; $\eta_{p}^{2}$ = .016, but neither of the other two scales showed interaction significant effects.

#### Processing speed difficulties

Participants were grouped into those with processing speed problems and students with no specific learning difficulties (control group). A two‐way mixed MANOVA did not show a significant effect on the change in COSQ scores, *F*(4, 306) = 0.45, *p* = .77; Wilk’s Λ = 0.99; $\eta_{p}^{2}$ = .006.

### Mental well‐being and COSQ

Our third hypothesis was that mental well‐being would be negatively associated with a reduction in concentration, engagement, ability to learn, and self‐worth due to greater difficulties with online learning. Pearson product–moment correlations were calculated between mental well‐being as measured by the SWEMWBS and the COSQ scores. Table 4 demonstrates significant associations between mental well‐being and all COSQ sub‐scales with medium to large effect sizes. For example, there was a .58 correlation between ability to learn in the online environment and mental well‐being. We further investigated change in COSQ scores due to online learning and the classroom environment with mental well‐being. The hypothesis of a reduction in mental well‐being due to difficulties with online learning was partially confirmed, with significant negative effects for concentration and ability to learn, with small effect sizes (Table 4).

**Table 4:** Correlations between changes in COSQ scores and mental well‐being (SWEMWBS)

Sub scales	*n*	Classroom	Online	Difference between classroom and online scores
Concentration	362	.42**	.46**	−.14*
Engagement	370	.38**	.42**	−.07
Ability to learn	370	.53**	.58**	−.10*
Self‐Worth from learning	369	.58**	.61**	−.07

** Statistically significant variables at *p* < 0.01.

* Statistically significant variables at *p* < .05.

## Discussion

### Online versus classroom‐based Learning

The results support our primary hypothesis that pupils’ self‐reported concentration, engagement, and ability to learn were significantly lower during online learning. These effects are ‘large’ by conventional standards (Cohen, 1988). Students’ perceived self‐worth from learning was also reduced by online learning with a medium effect size. The results support earlier arguments that online education is challenging for pupils and can impact learning (Friedman, 2020) and complements research from parental perspectives on a lack of pupils’ engagement and self‐worth when online learning (Garbe et al., 2020).

Pupils reported greater difficulty in the ability to concentrate during online lessons. Pupils reported more distractions by noise and devices during online learning, supporting Friedman’s (2020) research. It may be advisable for schools and parents/carers to find suitable solutions to improve monitoring of devices, such as mobile phones, game consoles, and other devices during online lessons to minimize distractions.

Student engagement with teachers is critical to learning (Furrer, Skinner, & Pitzer, 2014). Pupils develop perceptions about their ability to learn through student‐centred teaching, which involves reflection, interaction, and discussion (Barr & Tagg, 1995), which also links to personal development and academic performance (Pascarella & Terenzini, 2005). Future research should evaluate how online learning leads to less engagement and poorer perceptions of ability to learn, and what can be done to ameliorate this.

The period being studied was during the global COVID‐19 pandemic (March–July 2020). Educators were forced to deliver content in an unfamiliar context, direct to learners’ homes, via the unfamiliar means of online virtual learning platforms, previously only used for supplementary work, such as homework. As teachers adapted to delivering online, as learners became *au fait* with the technology, and as the providers of the online learning platforms improved functionality, the engagement and concentration of students may have improved. Further studies are needed to test these possibilities. Many schools may well choose to utilize online learning practices in future; however, this is likely to be without further school closures but through online methods that schools consider for the future.

A further aspect that could be investigated is perspectives of teachers in delivering online. Recent research by Nambiar (2020) addressed teachers’ perspectives of online learning during the pandemic and found that 37.1% of teachers reported low student involvement and engagement. They reported poor attendance due to connectivity issues, a lack of motivation, and difficulty assessing if the student’s understood the materials.

It is likely that some aspects of online learning will be integrated with classroom learning and assessing the effectiveness of this teaching is needed. This may include online whiteboards, whole‐class response systems such as quizzes, and other features of the electronic learning platforms that allow teachers to comment during real time. Teachers need to develop new skills to ensure their students' engagement and develop methods of preventing learners from circumventing their efforts.

### Relationship to specific learning difficulties

The results partially support our hypothesis that specific learning difficulties would relate to the magnitude of the decrease in engagement, concentration, self‐worth from learning, and perceived ability to learn. We were unable to demonstrate any significant differences between online and classroom‐based learning scores for pupils with processing speed impairments. However, pupils with working memory impairments show lower scores for online learning. At univariate level, there was a significant impact of online learning on concentration and engagement. This study demonstrates pupils with working memory problems were more affected by online learning, requiring additional support.

Working memory capacity varies widely, and individual differences in working memory capacity appear to have significant consequences for children’s ability to acquire knowledge and new skills in the classroom (Alloway et al., 2005). In speaking to pupils about their experiences of online learning, some reported missing vital parts of the lesson due to lack of attentional capacity. Pupils reported feeling self‐conscious drawing attention to this, leading to confusion. One proposed strategy was to watch recordings of lessons again to catch up. Delivery of a mix of synchronous and asynchronous material, thus allowing learners to actively learn and move through materials at their own pace.

Improvements in teachers’ ability to elicit feedback would assist them in assessing pupils' engagement and understanding and thus identify any steps in learning require repetition. Conversely, the ability of pupils to discreetly signal misunderstanding would help formative assessment for teachers. Just as teachers adapt delivery in the classroom, they need to differentiate delivery to suit individual learners’ needs when teaching online. As teachers become more skilled in utilizing online platforms, they will develop their repertoire of activities and pedagogical approaches to facilitate effective instruction.

### Mental well‐being

This study investigated students’ mental well‐being and their perceived level of engagement, concentration, self‐worth from learning, and the ability to learn. High self‐reported levels of concentration, engagement, ability to learn, and self‐worth through learning were associated with high mental well‐being levels. Importantly, the decrease in concentration and perceived ability to learn due to online education is associated with a decrease in pupils’ mental well‐being. Hence, poor learning environments negatively impact learning, engagement, and concentration and significantly impact children’s mental well‐being. Our findings are consistent with research highlighting higher engagement levels, namely dedication and vigour when learning, are related to better levels of well‐being (Cadime et al., 2016). As 50% of lifetime cases of mental health disorders begin before the age of 14 (Kessler et al., 2005), there have been concerns that the pandemic will lead to a surge of mental health difficulties for young people (Gray et al., 2020; Lee et al., 2020). The online environment has become a common method of education and will no doubt continue to be present for at least the short‐ to medium‐term within education. Given the demonstrated association between engagement in education and mental well‐being, it is imperative that educators find ways of overcoming problems encountered during online education to prevent a greater proportion of young people requiring mental health support and intervention.

Anxiety in pupils during sudden changes in working environments is well researched. Oyedotun (2020) found that fear and anxiety surfaced among students due to sudden changes from face‐to‐face learning to online learning. While such implications are unknown, there is potential for increased anxiety in the long term (Loades et al., 2020). This may be a concern that school staff and Educational Psychologists will need to consider going forward. The findings identified will help inform policy and practice for supporting adolescents’ learning and mental well‐being in the future during transitions of learning environments.

As the world begins to recover from the COVID‐19 pandemic and as learners return to their schools, school leaders should account for the adverse impacts on learning caused by being at home and plan for measures to ameliorate these negative effects. Factors such as poor health routines, changes to social relationships, and caring for siblings to allow parents to work (Kassa & Pavlopoulou, 2021) have all had potential negative contributions to learning and young people’s well‐being.

### Limitations and future research

There are several limitations to the current research. First, most participants were female. It is essential to evaluate if a similar pattern of results is obtained in a sample of male pupils. It is also important to consider the impact of online education across different ethnic groups and cultures within and outside of the United Kingdom and compare pupils with different levels of learning need, and different age groups.

Data were collected retrospectively, leading to difficulties in the reliance on memory. Future research may consider prospective designs as circumstances change. Future research may also consider the personal perspectives of young people through qualitative small focus groups to capture in‐depth personal accounts and experiences of online learning.

Other limitations included reliance of data generated by a new psychometric measure, leading to restriction in the ability to compare research with previous data directly. We hope future research may consider this measure and developing it further to provide comparison data and more information on its psychometric properties.

It is also important to consider whether the present findings are due to the novel sudden move to online teaching due to the impact of the pandemic. It is possible that, as both teachers and students learn from these early attempts to cope with online education, many of the problems highlighted by this research may resolve. It is also important to highlight that the primary objective of the sudden shift to online learning primary objective was not to re‐create a strong, robust educational ecosystem but rather a temporary shift of an alternate delivery mode that was quick to set up and was reliably available during school closures. Understanding this contrast to typical online learning which involves full‐time course development which may take months to create. Therefore, online learning in this manner is only accepted as temporary solutions to an immediate problem at hand.

Finally, while we wished to examine whether students with specific learning disabilities had particular problems with online learning, the study was constrained by the small number of students with specific learning needs. In particular, the sample size for those with a diagnosis of dyslexia was not deemed sufficiently large to provide an appropriate test of this hypothesis. Further research with larger sample sizes, or with a clearer focus on testing students with specific learning disabilities, is needed to provide a proper test of these hypotheses.

### Conclusion

The study shows a substantial decrease in pupil’s learning experiences (concentration, engagement, ability to learn, and self‐worth from learning) during online learning compared to their usual classroom experience that is associated with a decrease in mental health. The problem is exacerbated for those with working memory problems. More work is needed within this area to counter the implications individuals face due to online learning and, therefore, improve pupils' mental health.

## Funding

No funding was provided for this research.

## Conflict of interest

All authors declare no conflict of interest

## Author contributions

**Nicola S Gray:** Conceptualization (equal); Data curation (equal); Investigation (equal); Methodology (equal); Project administration (equal); Supervision (equal); Validation (equal); Visualization (equal); Writing – review & editing (equal). **Thomas Walters:** Conceptualization (equal); Investigation (equal); Methodology (equal); Software (equal); Writing – review & editing (equal). **Nicola J Simkiss:** Data curation (equal); Formal analysis (equal); Investigation (equal); Methodology (equal); Software (equal); Visualization (equal); Writing – original draft (equal). **Robert Snowden:** Data curation (equal); Formal analysis (equal); Supervision (equal); Validation (equal); Visualization (equal); Writing – review & editing (equal).

## Data Availability

The datasets used and/or analysed during the current study are available from the first or corresponding author on reasonable request.
